# Engineered resistance and risk assessment associated with insecticidal and weeds resistant transgenic cotton using wister rat model

**DOI:** 10.1038/s41598-022-06568-y

**Published:** 2022-02-15

**Authors:** Adnan Iqbal, Muhammad Azam Ali, Shafique Ahmed, Samina Hassan, Naila Shahid, Saira Azam, Abdul Qayyum Rao, Qurban Ali, Ahmad Ali Shahid

**Affiliations:** 1 Centre of Excellence in Molecular Biology, University of the Punjab, 87-West Canal Bank Road, Lahore, 53700 Pakistan; 2 Institute of Molecular Biology and Biotechnology (IMBB), Centre for Research in Molecular Medicine (CRIMM), University of Lahore, Lahore, Pakistan; 3 Department of Molecular Biology, Virtual University of Pakistan, Lahore, Pakistan; 4 Allied Health Sciences, The Superior College, Lahore, Pakistan; 5 Kinnaird College for Women University, Lahore, Pakistan

**Keywords:** Biotechnology, Risk factors

## Abstract

Stacking multiple genes into cotton crop to cop up multiple biotic stresses such as insects and weeds is a promising tool to save crop from losses. Transgenic cotton variety, VH-289, with double *Bt* and *cp4EPSPS* genes under the control of 35S promoter was used for the expression analyses and biosafety studies. The transgenic cotton plants were screened through PCR amplification of fragments, 1.7 kb for *Cry1Ac*, 582 bp for *Cry2A* and 250 bp for *cp4EPSPS*; which confirmed the presence of all genes transformed in transgenic cotton. The Cry1Ac + Cry2A and cp4EPSPS proteins were quantified through ELISA in transgenic cotton plants. The Glyphosate assay performed by spraying 1900 mL per acre of glyphosate Roundup further confirmed complete survival of transgenic cotton plants as compared to the non-transgenic cotton plants and all weeds. Similarly, insect infestation data determined that almost 99% insect mortality was observed in controlled field grown transgenic cotton plants as compared to the non-transgenic control plants. Evaluation of effect of temperature and soil nutrients availability on transgene expression in cotton plants was done at two different cotton growing regions, Multan and Lahore, Pakistan and results suggested that despite of higher temperature in Multan field, an increased level of Cry and cp4EPSPS proteins was recorded due to higher soil organic matter availability compared to Lahore field. Before commercialization of any transgenic variety its biosafety study is mandatory so, a 90 days biosafety study of the transgenic cotton plants with 40% transgenic cottonseeds in standard diet showed no harmful effect on wister rat model when studied for liver function, renal function and serum electrolyte.

## Introduction

Cotton is an important cash crop which significantly contributes to the economy of Pakistan. It meets the demands of local textile industry by providing the raw material, such as cotton lint to prepare fabrics for exports^1^. Beside the importance of cotton there are many threats for cotton such as insects, weeds and viruses. Lepidopteran insects pose enormous threat to the cotton crop. It has been reported that about 30% losses of cotton occur due to insect pests such as bollworms, spotted bollworm, pink bollworms and armyworm a devastating pathogen that result in significant losses and deteriorate cotton quality. As a result, cotton yield has reduced significantly^2^.

The other important threat to the cotton crop is weeds which affect the yield of cotton crops during first few weeks and multiple chemicals are used to control these weeds. Almost 40% losses in cotton are due to weeds^3,4^. With the limitations of current strategies to control insects and weeds and the lack of long-term control governed by applying different pre-emergence chemicals for weeds and chemical sprays for insects instigates the use of molecular biological tools like transgenic crop technology for gene pyramiding against different problems.

The chemical application to control the insects and weeds is harmful for environment that’s why the acceptable approach is to exploit the potential of insecticidal *Bacillus thuringiensis* through genetic engineering approach. Among *Bacillus thuringiensis* (*Bt*) genes such as *cry1Ac* + *cry2Ab*, *cry1Ac* + c*ry1F* or c*ry1Ac*, have been mainly used in genetically modified (GM) crops including cotton and maize^5–8^. There is no doubt that abiotic stresses, such as temperature, light, water scarcity, salinity, or nutrient deficiency, directly affect the quality and quantity of many crops production^9^. It has been reported that the expression of transgenes is affected by extreme environmental-factors in GM crops such as water stress and nitrogen deficiency affects the *Bt* maize^10,11^.

Chemical herbicides are used to control weeds but the differentiation between crop plants and weeds by herbicides is still a problem. Development of herbicide tolerant crops is the solution to this problem which is anticipated^4^. Synthetic herbicides have been used worldwide in agriculture to control weeds since 1940^12^. Registration of glyphosate-resistant soybean in (1996) and cotton in (1997) has made this technology for crops producers to accept more readily. Moreover, adaptation of Glyphosate resistant crop has generally reduced the opposing environmental and health influences of weed management^13^.

The first GM crop was introduced in 1994 and since then the concerns about the release of GM crops in the environment has increased tremendously^14^. Therefore, for the past few decades, the biosafety study of GM crops has become an area of intense interest for the idea of general acceptance of GM crops to consider them as safe to use as food and feed^15^. Risk assessment study is necessary before the release of any GM crop in environment for its commercial use and GM crop can only be considered as safe to use when, after risk assessment study, the GM crop is equivalent to the non-GM crop in terms genomic and phenotypic characteristics^16^.

In current study, a local transgenic cotton variety VH-289, harboring *cp4EPSPS*, *Cry1Ac and Cry2A* genes, was evaluated for protein expression and biosafety study. The effect of temperature, water and soil was also evaluated through plantation at two different cotton growing regions i-e Multan and Lahore. Local crop verities are preferred to use for the development of transgenic crops as they are best suited to the local environmental conditions. VH-289 is a local cotton variety. This variety was selected for the study as it has 95% germination rate and out of germinated embryos 98% plant formation rate which one of the highest germination and plant formation rates among various local cotton accessions^17^.

## Materials and methods

### Acquisition of Transgenic seed

The Transgenic cotton seeds were acquired from the Plant Biotechnology Lab of Center of Excellence in Molecular Biology, University of the Punjab, Lahore, Pakistan. It has been confirmed that the experimental samples of plants, including the collection of plant material, complied with relevant institutional, national, and international guidelines and legislation with appropriate permissions from authorities of Centre of Excellence in Molecular Biology, University of the Punjab, Lahore, Pakistan for collection of plant specimens. All methods for using rats in experiments were carried out in accordance with relevant guidelines and regulations of Centre of Excellence in Molecular Biology, University of the Punjab, Lahore, Pakistan and ARRIVE guidelines.

### Molecular confirmation of transgenic cotton plants

The experimental protocol was approved by Centre of Excellence in Molecular Biology, University of the Punjab, Lahore, Pakistan which are also similar as American Veterinary Medical Association (AVMA) Guidelines. Genomic DNA was isolated from leaves of transgenic cotton plants by following the same procedure^18^ with some modifications and used for amplification through PCR by using gene specific primers of *Cry1Ac*, *Cry2A* and *cp4EPSPS*: forward F-5′CATGGACAACAACCCAAACA-3′, & R-5′TTACCGAGTGAAGATGTAAAAGCA-3′ for *Cry1AC*, F-5′GAAGGAGTGGATGGAGTGGA-3′ & R-5′GCGGTCTGGTAGGTGTTGAT-3′ for *Cry 2A*, F-5′CGTGGGTGTGTATGACTTCG-3′ & and R-5′GTGTTGAGACCAGCGAGGAG-3′ for *cp4EPSPS*.

### Soil nutrients, water composition and temperature analyses

The advanced generations of transgenic cotton plants of VH-289 were grown at two cotton growing regions i-e Multan and Lahore to compare the impact of abiotic factors such as soil nutrients, water composition and temperature on transgenes expression at protein level. The data regarding irrigation water and soil composition of both locations was analyzed from laboratory testing facility of agriculture department government of Punjab Pakistan (http://www.agripunjab.gov.pk/labs), while temperature was recorded from the official website of AccuWeather (https://www.accuweather.com/).

### Expression analysis of transgenic cotton plants

Bradford assay^19^ was used for the quantification of total crude protein from young leaves of transgenic cotton plants of VH-289. The value of each sample was determined with the help of spectrophotometer (Bradford 1976). Protein expression of *Cry1Ac*, *Cry2A* and *cp4EPSPS* was quantified through Enzyme Linked Immunosorbent Assay, (ELISA) using Envirologix Kit (Cat # 051) according to procedure provided by the manufacturer and quantification of proteins was done as ng per gram of fresh tissue weight.

### Temporal Bt and cp4EPSPS protein expression in transgenic cotton plants grown at different locations

The leave samples of transgenic and control cotton plants were collected from two locations Multan and Lahore in the two inner rows/replicate plots. Terminal leaves from the middle part of the plants were taken at 15 days interval starting from 15 days after sowing (DAS) upto 120 days after sowing of transgenic cotton plants from both locations. The collected samples were stored in liquid nitrogen and brought to the laboratory for protein extraction. The crisped-dried samples from three plants per plant were placed in a 1.5 mL tubes. Quantification of Cry1Ac, Cry2A and cp4EPSPS protein was done using method available with enzyme-linked immunosorbent assay (ELISA) kits (EnviroLogix). Five milligrams ground samples were processed for analysis. Absorbance readings of the samples were taken at 595 nm**.** A correlation of Bt protein expression with vegetative and reproductive stages of transgenic cotton crops at different temperature and time was calculated in this way.

### Insect bioassay of Bt protein

The endotoxins efficacy against targeted insect pest i-e American Bollworms *Heliothis armigera* (2nd instar) larvae was evaluated through laboratory biotoxicity assays. The 60 days old leaves from upper, middle and lower portion of transgenic cotton plants were taken and placed in perti plate containing moist filter papers. Three 2nd instar larvae of Heliothis were subjected to each leave for 3 days. Mortality rate was observed and recorded after 2–3 days by the following formula:

$$\% {\text{Mortality}} = \frac{{{\text{No.}}\;{\text{of}}\;{\text{dead}}\;{\text{larvae}}}}{{{\text{Total}}\;{\text{no.}}\;{\text{of}}\;{\text{larvae}}}} \times 100$$

### Herbicide spray assay

For the evaluation of expression of cp4EPSPS gene, leaves of transgenic cotton plants were subjected to glyphosate spray assay at both locations respectively. Roundup™, commercially available herbicide Glyphosate, was used for this assay. Expanded leaves of 45 days old field grown transgenic and control cotton plants, at both locations (Multan and Lahore), were sprayed in concentration 1900 ml/80 L water/0.404 hac. Transgenic cotton plants and control plants were grown in separate lines and the effect of glyphosate spray on weeds and transgenic along with control cotton plants were observed regularly up to 15 days as described by^3^.

### Liver function test (LFT), renal function test (RFT) and serum electrolyte test for biosafety studies

To determine the effect of potential unintended effects of transgenic proteins, an additional animal study using wister rat model was conducted. Briefly, 30 male rats of about 5–6 weeks age were selected and randomly divided into three groups namely G1, G2 and G3. Rats in group G1 were reared on standard diet while rats in G2 and G3 were respectively reared on a diet containing non-transgenic and 40% of transgenic cotton seeds (expressing Cy1Ac, Cry2A and cp4EPSPS) or it’s near isogenic one. Animals were kept for 90 days under standard housing conditions^20^. At the end of the study blood samples were collected from all the animal groups in a serum separator tubes and sent to university of veterinary and animal science (diagnostic lab) for liver function test (LFT), renal function test (RFT) and serum electrolyte.

### Statistical analysis

Statistical analysis were performed using Ghaphpad Prism 7.0 software. Asterisks, *, **, *** and **** were used for denoting statistically significant values as *p-values* ≤ 0.05, 0.01, 0.001 and 0.0001 respectively for the comparison between values of transgenic and non-transgenic control, while “ns” symbol was used for indicating non-significant values.

## Results

### Molecular analysis of transgenic cotton plants

**Figure 1:**
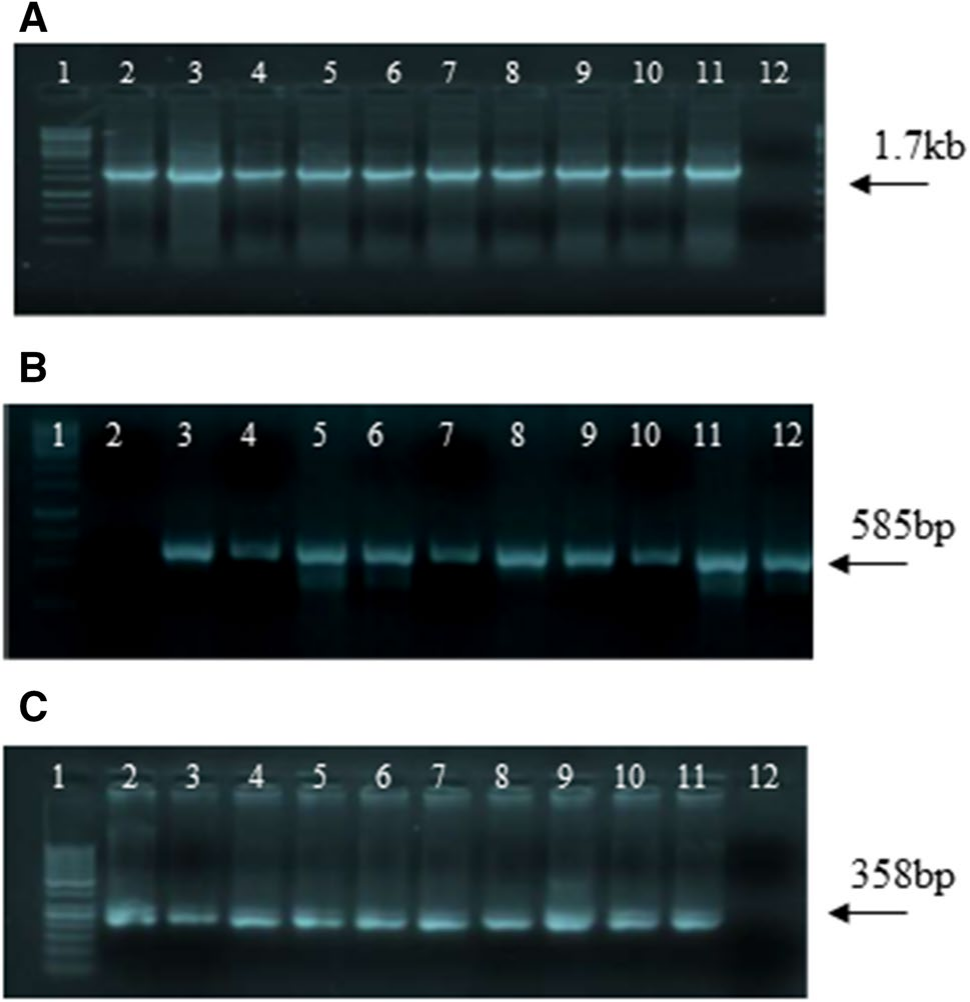
PCR confirmation of transgenic plants of T_0_: (**A**) Transgenic plants harboring Cry1Ac; Lane 1: 1-kb Ladder, Lane 2: Positive Control, Lane 3–11: Transgenic cotton plants, Lane 12: Negative control; (**B**) Transgenic plants harboring Cry2A; Lane 1: 1 kb ladder, Lane 2: Negative control, Lane 3–11: Transgenic cotton plants, Lane 12: 1-kb Ladder; (**C**) Transgenic plants harboring cp4EPSPS; Lane 1: 50 bp Ladder, Lane 2–10: Transgenic plants, Lane 11: Positive control, Lane 12: Negative control.

Nine transgenic plants were confirmed for the amplification DNA fragments of 1.7 kb, 585 bp and 358 bp for Cry1Ac, Cry2A and cp4EPSPS respectively (Fig. 1A–C) from advanced generation which was grown at two different field locations Multan field and Lahore field (See supplementary file for figure 1 A-C).

### Soil, water and temperature analyses

**Figure 2:**
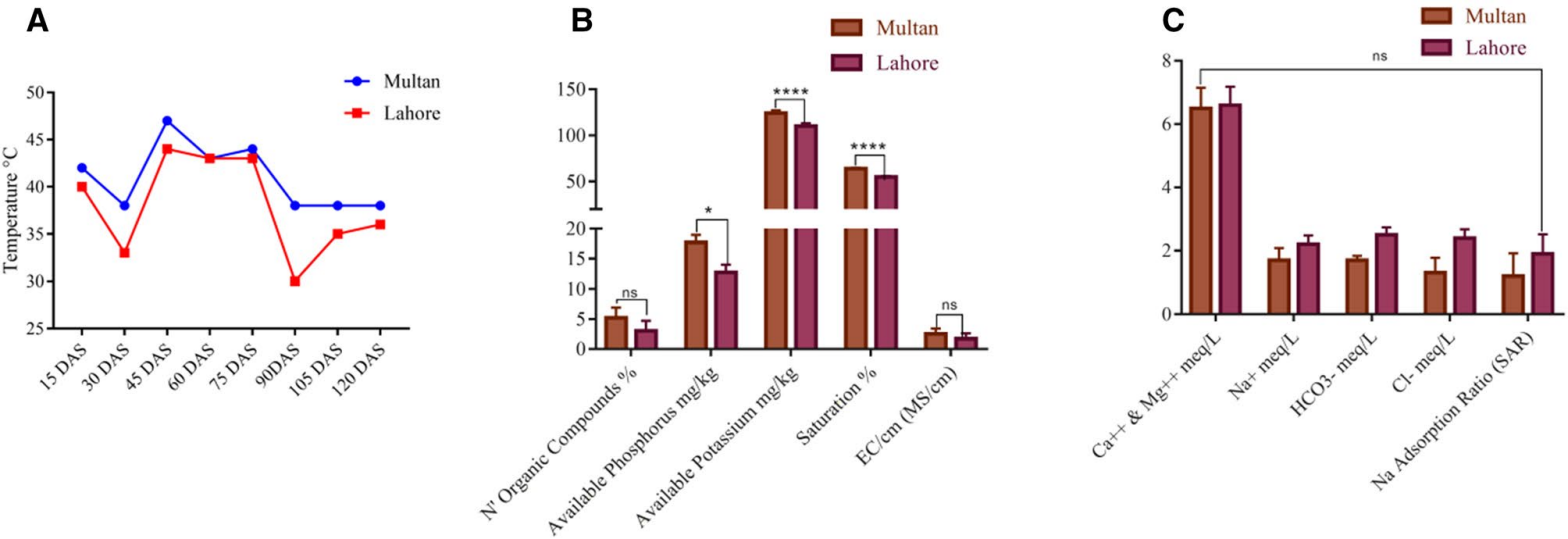
Graphical representation of temperature, soil nutrients and water analyses (**A**) Temperature data of Multan and Lahore fields (**B**) Soil Nutrients (**C**) Water analysis used for irrigation.

Before starting expression analysis, data of temperature was recorded from Multan and Lahore fields. Multan region found to be 2 °C to 3 °C hotter than Lahore (Fig. 2A). The results of soil analysis showed that the organic matters, available phosphorus, available potassium and soil saturation percentage were higher in Multan soil as 24%, 22.39 mg/kg, 14.93 mg/kg and 5.3% respectively as compared to Lahore soil components (Fig. 2B), while water analysis revealed that both areas water was favorable for irrigation considering the water quality guidelines for irrigation in Pakistan^21^ (Fig. 2C).

### Temporal expression of Bt and cp4EPSPS protein

**Figure 3:**
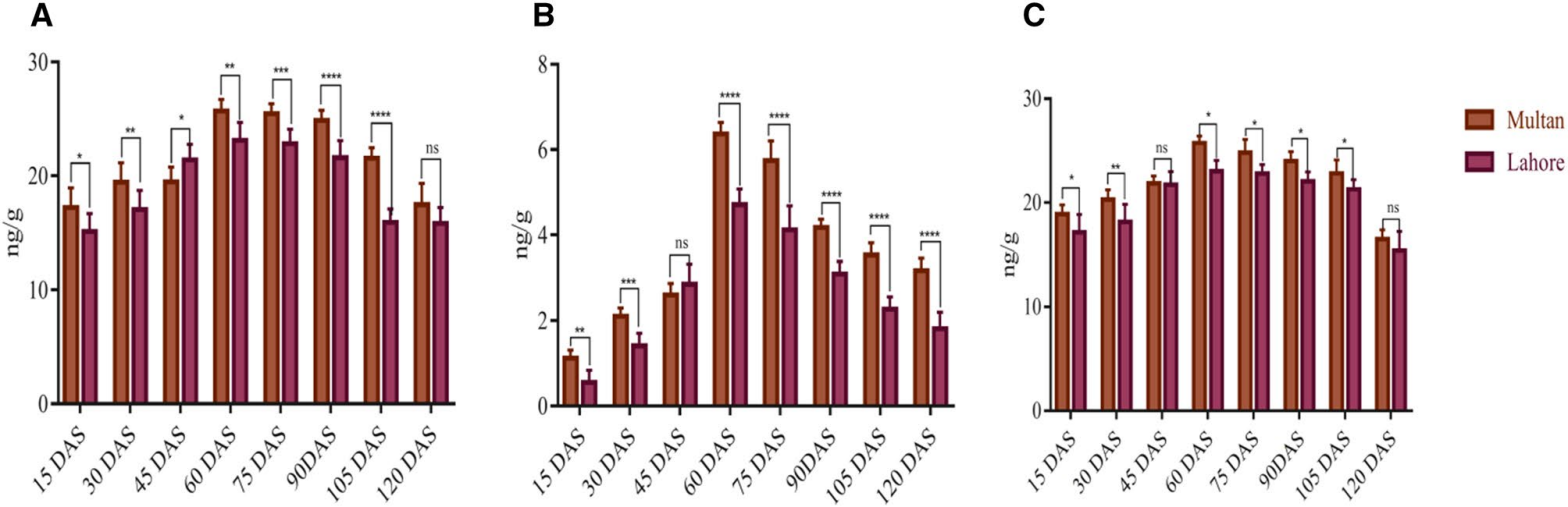
Graphs showing comparative expression of Cry and cp4EPSPS proteins of transgenic plants in Multan and Lahore fields. (**A**) Represents the expression of Cry1Ac in PCR confirmed transgenic plants at different time intervals respectively (**B**) Represents Cry2a in PCR confirmed transgenic plants at different time intervals respectively (**C**) Represents cp4EPSPS PCR in confirmed transgenic plants at different time intervals respectively. Each bar represents the average value of 9 selected lines from both Multan and Lahore field.

Correlation analysis for the evaluation of Cry and cp4EPSPS protein expression, sampling from T2 generation after every 15 days intervals for at-least 120 days after the date of sowing from cotton plants experimental area, Multan and from Lahore fields displayed variations. A higher trends of protein values of Cry1Ac, Cry2A and cp4EPSPS were recorded in cotton plant samples taken from Multan field as compared to Lahore field (Fig. 3 A–C).

### Insect bioassay

**Figure 4:**
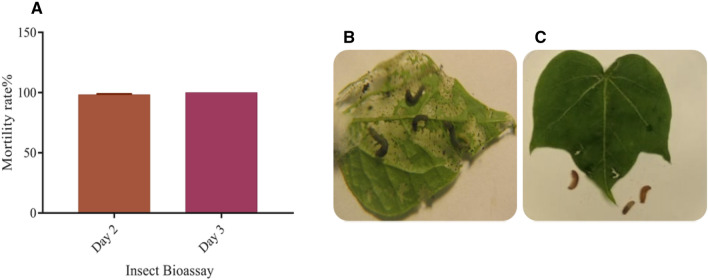
Insect bioassay (**A**) Graphical representation of insect bioassay mortality data on transgenic cotton plant leaves at day 2 and day 3 (**B**) Represents the control non-transgenic leaf highly damaged by alive and healthy insects and also increased in size due to constant feeding (**C**) Represents the non-damaged transgenic cotton plant leaf with dead insects along with no growth of insect in size.

Lepidopteran insect bioassay was used to check the efficacy of *Bt* genes against American Bollworms *Heliothis* larvae (*2nd instar*) in transgenic cotton plants. Mortality rate was evaluated after 2–3 days of insect assay on fresh cotton leaves of transgenic and non-transgenic control plants placed on wet filter paper in petri plates. On 2nd day 98.5 and 3rd day a 100% mortality rate of Heliothus larvae was found on transgenic cotton plants leaves with almost no damaged to leaves as compared to non-transgenic cotton plant leaves which were heavily damaged by bollworms larvae and the at third day and almost completely damaged at fifth day of insect assay (Fig. 4A–C).

### Herbicide spray assay

**Figure 5:**
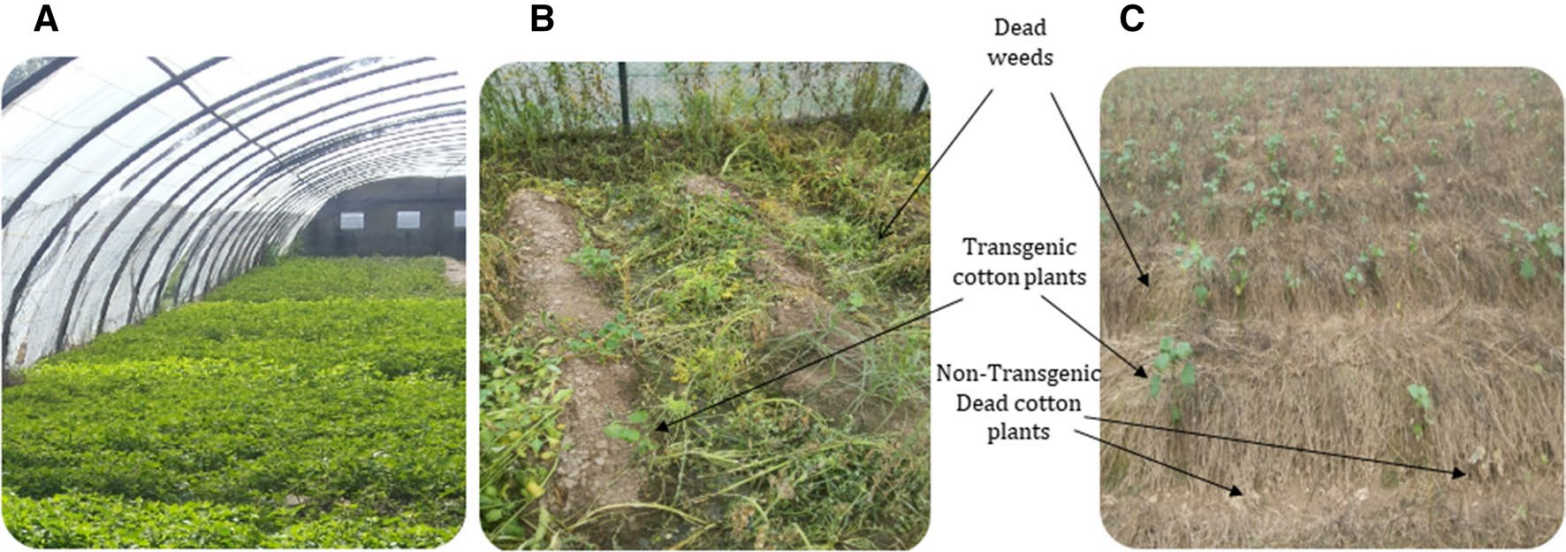
Glyphosate spray assay of transgenic cotton plants at day 3 and 5 (**A**) Transgenic cotton plants along with weeds before spray, (**B**) View of cotton field after 3 days of glyphosate spray, arrow indicate the necrotic weeds and survival of transgenic cotton plants (**C**) View of cotton field after 5 days of glyphosate spray, arrow indicate the complete mortality of weeds and survival of transgenic cotton plants.

The efficacy of cp4EPSPSgene in transgenic cotton plants was confirmed through Glyphosate spray assay. Initially, the transgenic cotton plants in fields, compared to non-transgenic control plants and weeds, showed no effect of glyphosate spray (1900/acre) however, necrosis started at third day and leads to complete death of non-transgenic plants along-with weeds until fifth day (Fig. 5A–C).

### Liver function test (LFT), renal function test (RFT) and serum electrolyte

The results of liver function test (LFT), renal function test (RFT) and serum electrolyte are tabularized in Table 1. The resulted presented suggests no significant difference between G2 and G3 when compared to the G1 (control) group of rats (Tables 1, 2 and 3).

**Table 1:** Liver function tests.

Parameters	Standard diet control (G1)	Non-GM (G2) Diet 30%	GM (G3) Diet 40%	PV
Total Bilirubin (mg/dl)	0.65 ± 0.05	0.65 ± 0.05	0.6 ± 0.00	0.6495
Alanine Aminotransferase (U/L)	212.5 ± 85.500	242.5 ± 25.50	261 ± 34.00	0.05591
Aspartate Aminotransferase (U/L)	225.5 ± 183.500	243.5 ± 9.50	248 ± 40.00	0.3198
Alkaline Phosphatase (U/L)	944 ± 358.00	974.5 ± 59.50	961.5 ± 141.50	0.2833

**Table 2:** Renal function tests.

Parameters	Standard diet control (G1)	Non-GM (G2) Diet 30%	GM (G3) Diet 40%	PV
Urea (mg/dl)	24 ± 11.00	30 ± 3.00	29 ± 0.00	0.4231
Creatinine (mg/dl)	0.65 ± 0.05	0.5 ± 0.100	0.5 ± 0.00	0.3065

**Table 3:** Serum electrolytes.

Parameters	Standard diet control (G1)	Non-GM (G2) Diet 30%	GM (G3) Diet 40%	PV
Na (mEq/L)	127.5 ± 1.500	122.5 ± 2.500	125.5 ± 0.500	0.2611
K (mEq/L)	17.45 ± 1.450	15.9 ± 0.300	15.3 ± 2.300	0.6527
Cl^-^ (mmol/L)	107.35 ± 3.350	112 ± 1.00	110 ± 1.00	0.3015

## Discussion

Cotton as a backbone of Pakistan’s economy has been facing many challenges in the form of insect pest and weeds. According to an estimate upto 40% of the total losses of cotton crop is due to the weeds, 30% losses occur due to insect pest which are also major limiting factor for various other economically important crops^2,4,22^. In past insects were controlled by spraying pesticide and the expenditure alone in USA has reached to 40 billion USD annually^23^. Biotechnology holds promising solution for these types of problems. The use of genes responsible for adequate quantity production of toxin cry protein for insects and alternate EPSPS production through genetic modification of cotton with synthetic cp4EPSPS gene can be the possible measures against such type of problems opted in current study.

In current study the effort has been made to evaluate the protein expression and conduct biosafety study of transgenic cotton variety VH-289 harboring two insecticidal genes *Cry1Ac* and *Cry2A* genes in combination with a weedicide *cp4EPSPS* gene and to find out the effect of environmental factor such as temperature, soil nutrients and water quality on gene expression level. Based on the background genetically modification of cotton plants for resistance against insects and weeds through incorporation of the *Cry1Ac* & *Cry2A* and *cp4EPSPS* genes into Pakistan’s local variety of cotton has been cemented.

Molecular approaches such as PCR were used for confirmation of transgene in advanced generation of cotton variety VH-289 as was done by^17,24^. Transgenic cotton plants successfully amplified the DNA fragments of 1.7 kb, 585 bp and 358 bp for *Cry1Ac*, *Cry2A* and *cp4EPSPS* respectively (Fig. 1). To Different transgenic cotton plants showed varied concentration of Cry1Ac, Cry2A and cp4EPSPS proteins at different time intervals when subjected to temporal expression as was the case with Bakhsh et al., (2010) having variable temporal expression of Cry proteins in different transgenic cotton plants. The protein expression for both Cry and cp4EPSPS gradually increased from 15 DAS up to 60 DAS and started decreasing until 120 DAS. The maximum expression of Cry1Ac, Cry2A and cp4EPSPS was recorded at 60 DAP where Cry1A was found to be 25.6 ng/g and 23.11 ng/g, Cry2A 6.37 ng/g and 4.71 ng/g and cp4EPSPS 25.73 ng/g and 23. 03 ng/g in transgenic plants of Multan and Lahore respectively (Fig. 3A–C).

As the expression of *Bt* genes vary with temperature as described by Chen et al., (2005), where expression of *Cry1Ac* gene significantly reduced at 37 °C but in contrast to this study, Multan region which is on average 2 °C to 3 °C hotter than Lahore as per data recorded (Fig. 2A), it was found that protein expression of Cry1Ac, Cry2A and cp4EPSP in Multan region was higher than Lahore region right from the beginning till end of the growing season (Fig. 3A–C) this higher protein expression can be attributed to the higher soil organic matters, phosphorus, potassium and saturation level in Multan field as 24%, 22.39 mg/kg, 14.93 mg/kg and 5.3% respectively as compared to soil of Lahore field (Fig. 2B). Similar results were reported by^25^ they got elevated level of Bt endotoxin expression by 14% on increasing dose of N fertilizer.

The bio-toxicity leaf assay was used to evaluate the efficiency of the transgenic proteins Cry1Ac and Cry2A in cotton plants compared to non-transgenic control plants using *Heliothis* (2nd instar larvae). The larvae were freed in each petri-plate with a transgenic cotton plant leaf, along with a control, non-transgenic cotton plant in a separate plate, died completely at day 3 of bio-toxicity assay (Fig. 4A). These results are in accordance with the results reported by^26^. The study of transgenic endotoxin protein level is very crucial, as it must be in a sufficient quantity at the time of insect infestation to protect the crop against lepidopterons, especially the boll worms^27^. A 100% insect mortality was recorded in petri plate of transgenic cotton plants compared to non-transgenic control cotton leaves where insects survived and grow with passage of time. Similar results were obtained by^27^.

A glyphosate-herbicide (1900 mL/acre) was sprayed to the field growing transgenic cotton plants to determine the herbicide resistance. The non-transgenic control cotton plants along-with weeds first showed the necrotic-spot and finally started dying as compare to the healthy transgenic plants (Fig. 5). These results are parallel with other similar studies including^28^. In view of the above results, it can be concluded that transgenic cotton plants that contain the *cp4EPSPS* gene have considerable resistance against weedicide.

Before release of any GM crop in the environment for its commercial use it is very important to evaluate it for its any possible toxicity, therefore the biosafety study of GM crops has become area of interest for the past few decades^29^. The cottonseed are used as a protein source for the livestock and also used as a rich source of vegetable oil. Biosafety studies of many transgenic plants expressing insecticidal protein such as Cry1Ac and Cry2A especially in cotton plants has been done thoroughly^30,31^. This study was designed to evaluate transgenic cotton variety VH-289, containing *Cry1Ac*, *Cry2A* and *cp4EPSPS* for its expression analysis and biosafety study for the safe release in environments. Similar studies have also been reported previously^32–34^. It has been proven by the previous studies that the Cry1Ac and Cry2A proteins are safe to use in GM crops and cause no harm to the mammals because of their acidic environments in stomach^35^. Our biosafety study on wister rats, which involved using 40% GM cottonseed as a diet in (G3) group and 30% Non-GM cottonseed as diet in (G2) group, showed no comparable change of harmful effect on LFT, RFT and serum electrolyte when compared to the standard diet control group (G1). These results are consistent with the previous studies^20,36^.

The study utilizes the novel idea of co-expression of three gene including two Bt genes and one weedicide resistant gene for the exploration of the factoring affecting their expression in local cotton variety VH-289. The expression of Cry1Ac and Cry2A in VH-289 has been found very effective against chewing insects (Fig. 4A–C) and the results of expression of cp4EPSP has also been very encoring for the control of weeds (Fig. 5A–C). Previous studies have shown that the success of the control of chewing insects including cotton bollworm, pink bollworm, spotted bollworm and armyworm depends on constantly higher expression of transgenes^27,37^. Similarly, higher expression of weedicide resistant gene (cp4EPSPS) is necessary for the effective control of weeds^3^. The study has also given the insight into exploring the external factors such as temperature, soil nutrients and water quality on the transgenic cotton variety VH-289. The study also evaluated the risk assessment associated with the locally developed transgenic variety VH-289 and concluded that no harmful effects were found on the wister rat model (Tables 1, 2, 3) and it is the safe to release for the commercial use in the field.

## Supplementary Information

Supplementary Information.
