# Supplementary material for: Engineered resistance and risk assessment associated with insecticidal and weeds resistant transgenic cotton using wister rat model
Source: Sci Rep. 2022 Feb 15;12:2518. doi: 10.1038/s41598-022-06568-y (PMC8847412; doi:10.1038/s41598-022-06568-y)
Supplement: Supplementary file 1 — Supplementary Information. [file 41598_2022_6568_MOESM1_ESM.docx]

**Orginal Gel pics**

## Engineered resistance and risk assessment associated with insecticidal and weeds resistant transgenic cotton using wister rat model

***Adnan Iqbal^1, 2^, Muhammad Azam Ali^3^, Shafique Ahmed^4^, Samina Hassan^5^, Naila Shahid^1^, Saira Azam^1^, Abdul Qayyum Rao^1^, *Qurban Ali^2^ and Ahmad Ali Shahid^1^**

*^1^Centre of Excellence in Molecular Biology, University of the Punjab, 87-West Canal Bank Road, Lahore, 53700, Pakistan*

*^2^Institute of Molecular Biology and Biotechnology (IMBB), Centre for Research in Molecular Medicine (CRIMM), University of Lahore, Lahore, Pakistan*

*^3^Department of Molecular Biology, Virtual University of Pakistan-Pakistan*

*^4^Allied Health Sciences, The Superior College, Lahore, Pakistan*

*^5^Kinnaird College for Women University, Lahore, Pakistan*

*Corresponding author email:* [*saim1692@gmail.com*](mailto:saim1692@gmail.com)*,* [*aadiiq@yahoo.com*](mailto:aadiiq@yahoo.com)


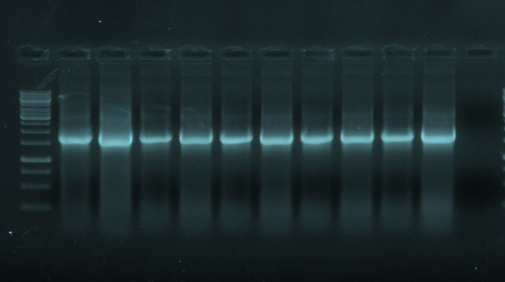


A


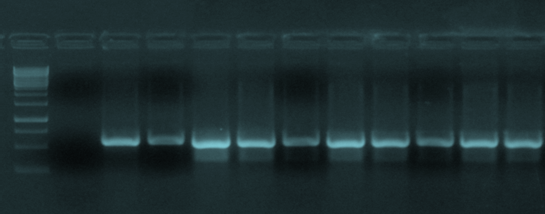


B


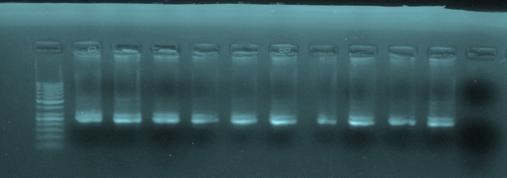


C

**Figure 1: PCR confirmation of transgenic plants of T_0_: (A)** Transgenic plants harboring Cry1Ac; Lane 1: 1-kb Ladder, Lane 2: Positive Control, Lane 3-11: Transgenic cotton plants, Lane 12: Negative control; **(B)** Transgenic plants harboring Cry2A; Lane 1: 1kb ladder, Lane 2: Negative control, Lane 3-11: Transgenic cotton plants, Lane 12: 1-kb Ladder; **(C)** Transgenic plants harboring cp4EPSPS; Lane 1: 50bp Ladder, Lane 2-10: Transgenic plants, Lane 11: Positive control, Lane 12: Negative control
